# Do patients’ resilience and subjective illness representation predict the outcome of a routine inpatient treatment program of major depressive disorder?

**DOI:** 10.1007/s00406-021-01285-5

**Published:** 2021-06-30

**Authors:** Laura Marschollek, Udo Bonnet

**Affiliations:** 1 Department of Psychiatry, Psychotherapy and Psychosomatic Medicine, Evangelisches Krankenhaus Castrop-Rauxel, Grutholzallee 21, 44577 Castrop-Rauxel, Germany; 2 Department of Psychiatry and Psychotherapy, Faculty of Medicine, LVR-Hospital Essen, University of Duisburg-Essen, Essen, Germany

**Keywords:** Self-regulation, Personal resources, Trust in doctors, Affective disorders, Real-world treatment, Early sudden gain

## Abstract

Adopting a personalized medicine approach beyond genetic/epigenetic profiling within psychiatric diagnostic and treatment is challenging. For the first time, we studied the influence of two patient resources (resilience and illness representation) on the success of an inpatient treatment of major depressive disorder (MDD). Using a 5-week observational real-world-study, the treatment- success was measured by the difference between the subjective depression- severity (according to the German short form of Beck’s Depression-Inventory) at baseline (i.e., days four to six post-admission) and study- endpoint. In the intention-to-treat sample (*n* = 60, 47.3 ± 12.8 years old; 58% females), the patients’ illness representation [measured by the “Krankheitskonzeptskala” (KK)] did not predict their treatment- success. The KK-dimension ‘trust-in-doctors’ was associated with resilience but not with the treatment-success. Albeit, the patients’ resilience (determined by Resilience- Scale, 11-item-version (RS-11)) negatively predicted their positive treatment- success (*b* = − 0.09, *p* = 0.017, *f*^2^ = 0.11). This influence of resilience on treatment- success was completely mediated by the baseline-depression- severity. This means, patients with low resilience reported high baseline-depression- levels which predicted a significant positive treatment- success. And, patients with high resilience reported low baseline-depression-levels which predicted no relevant or even negative inpatient treatment-success. The latter “high-resilience”- group (*n* = 27) was especially interesting. Remarkably, these patients appeared to have experienced within the first four-to-six inpatient treatment-days an “early sudden gain” against their considerable MDD- burden that initially had led to their admission. Thus, a stronger resilience might serve as a proxy of the development of an early MDD-relief as well as of lower baseline-depression- levels. Further studies are warranted to support the value of a patient’s resilience to predict his treatment response and inpatient treatment duration.

**Supplementary Information:**

The online version contains supplementary material available at 10.1007/s00406-021-01285-5.

## Introduction

As well as in other Western countries, the major depressive disorder (MDD) belongs to the three most prevalent mental disorders also in Germany [1, 2]. In our country, among MDD-treatment-seeking persons, approximately 50% and 15% are in outpatient and inpatient/day patient settings, respectively [3]. Lower treatment motivation/expectation and lower patient satisfaction with psychiatric treatment as well as the number of comorbidities have been found to negatively predict the outcome of an inpatient MDD- treatment [4–6]. Studies inquiring patients’ resources as a potential catalyst for treatment effectiveness are very scarce. Therefore, we attempted to investigate whether two typical personal resources, i.e., the patient’s resilience [7–9] as well as illness representation [10, 11], could have an impact on the outcome (treatment success) of his inpatient MDD- treatment.

Why we have chosen these two personal resources? High resilience, i.e., the pronounced ability to adapt and thrive in the face of adversity [8], was found to mediate the positive treatment-success of mental illness, e.g., demonstrated in the outpatient treatment of MDD [12, 13]. An optimistic appraisal style seems to belong to the psychobiological key mechanisms of resilience itself [8, 9]. Furthermore, optimism might influence a person’s subjective illness representation [10, 11], here referred to as “concept-of-illness” [14]. The concept-of-illness-approach comprises a person’s attitudes, interpretations, explanations, and predictions towards his illness [14]. Resilience and concept-of-illness are related to the representation of Oneself (self-concept) [15], which is assumed to be at the very core of dimensional manifestations of mental disorders [16–18] by some authors [19]. In this overarching context, the questions arise (i) whether resilience and concept-of-illness actually interact with each other and (ii) to what extent both contribute to the inpatient treatment of MDD. To our knowledge, both subjects are awaiting first study, which we carried out as described below.

## Methods

### Study design and regimen

This single-center prospective observational study was conducted from April to August 2019 in a psychiatric ward (specialized in the routine treatment of depression) of the Evangelisches Krankenhaus Castrop-Rauxel near Dortmund, Germany. It was conducted in accordance with the revised declaration of Helsinki. The study was reviewed and approved by the Ethics committee of the Medical Faculty of the University of Duisburg-Essen, Germany. Within the first 5 days after admission, all patients were visited and asked to participate in the study (*eligibility verification*).

The study was scheduled for up to 5 weeks and comprised two measurement points: the first within 7 days post-admission (*baseline*) and the other within 7 days before discharge from inpatient treatment as usual of MDD (*study endpoint*). However, patients could be discharged earlier based on a shared patient/staff decision, when both parties agreed that the patient’s psychiatric and somatic condition had improved to the point that primary or secondary care would be sufficient for continuation treatment.

The inpatient treatment as usual of MDD consisted of antidepressant pharmacotherapy, psychotherapy, movement and occupational approaches, social counseling, psychiatric nursing, milieu therapy as well as identification and treatment of comorbidity. The inpatient face-to-face psychotherapy based on multimodal individual and group- psychotherapy (cognitive- behavioral, psychodynamic and humanistic elements including psychoeducation).

### Participants, eligibility and drop-out- criteria

Adult (18- to 65-year-old) patients with moderate (F32.1, F33.1) or severe unipolar MDD (F32.2, F33.2) according to ICD-10 who had sought inpatient treatment were eligible. A prior outpatient treatment attempt must have been unsuccessful. Additional *inclusion criteria*: participants must be familiar with the German language, did not have a lifetime diagnosis of schizophrenia or bipolar disorder, and were currently not psychotic or misusing alcohol or drugs. Their Mini-Mental-Status-Test must have revealed above 24 points and they had to provide their written informed consent to participate in this study**.** *Excluded* were patients who did not give back the baseline self-report questionnaire. Furthermore, those patients were excluded who attempted suicide throughout the study. Also excluded were patients using alcohol or drugs for recreational purposes. The same applied to patients who became psychotic or confused for longer than a 48 h-period or were transferred to another department (e.g., due to somatic complications) for longer than a 7-day-period. *Drop-outs* were defined as those patients who terminated the inpatient treatment prematurely, withdrew their consent to participate, lost-to-follow up or were excluded during the study.

### Measurements and primary outcome

We assessed the subjective MDD- severity via the German short (fast screen) form of Beck’s Depression Inventory (BDI-FS) [20] at baseline and study endpoint. All other measures were carried out only at baseline. At this time, the *resilience* was determined per Resilience Scale 11 (RS-11; a reliable 11-item, German short version of the Resilience Scale by Wagnild and Young [21]), and the *subjective illness representation* (*concept-of-illness)* was measured per “Krankheitskonzeptskala” [14]. All scales were self-reports and had been well validated [14, 20–22]. The *primary study outcome* was defined by the *treatment- success*, as determined by the differences between the BDI-FS- scores at baseline and at study endpoint. Per definition, the treatment- success could be positive or negative (see legend of Supplemental Table 2).

### Individual scales and their internal consistency in the present study

The BDI-FS comprises seven items that are rated on a four-point scale. This instrument achieved Cronbach’s *α*-values of 0.88 at baseline and.0.87 at study endpoint. According to [20], BDI-FS scores of 4–6 indicated a mild depression, scores of 7–9 a moderate and scores above 9 a severe depression. As shown by Poole et al. [22], the BDI-FS represents a good, simple and time-sparing alternative to the more commonly used BDI-II [20]. The RS-11 comprises 11 items, each rated on a seven-point scale, and was conceptualized as unidimensional. We found an excellent Cronbach’s α of 0.90. The Concept-of-Illness-Scale (Krankheitskonzeptskala) was developed and validated by Linden et al. [14]. This scale comprises 7 dimensions *(trust in medication, trust in doctors, guilt, negative expectations, control of coincidence, predisposition, and idiosyncratic beliefs*). Its sum-score included 29 items, each rated on a five-point scale [14]. The orientation of the subjective illness representation depends on the score: the higher the amount of the collected sum-points the more positive was a person´s concept-of-illness. [14]. Cronbach’s α of the Krankheitskonzeptskala was 0.66 which is acceptable for a multi-dimensional test-structure [23].

### Data collection

**Fig. 1:**
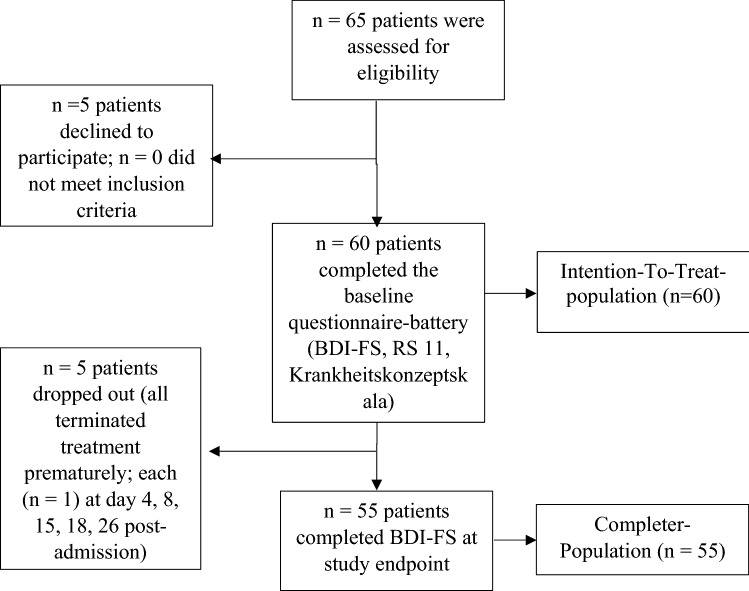
Enrollment of study participants

All data were collected by L.M. At baseline and study endpoint, measurements were carried out within 7 days post-admission and 7 days pre-discharge, respectively. This period should enable the participants to have enough time to complete the questionnaires. The baseline visit included distributing BDI-FS, RS-11, and the Krankeitskonzeptskala. The visit at study endpoint comprised completing BDI-FS again (Fig. 1).

### Sample size

Sample size was determined by G*Power [24]. The test family was *t*- test and the statistical procedure was linear multiple regression. We chose an effect size of Cohen’s *f*^*2*^ = 0.15 (low to medium) because in literature, the most correlation coefficients described a low to medium strength (effect) of the relationship between resilience and psychiatric symptoms, e.g., [25, 26]. We set the alpha level to the standard of 0.05 and the power to 0.85. This resulted in a required sample size of *n* = 50.

### Statistical analyses

We performed an intention-to-treat-analysis comprising all included patients. We did not need to modify the intention-to-treat-analysis because all included patients returned the battery of baseline tests. We used multiple imputation for handling missing data [27]. For all variables, we computed descriptive statistics (means, standard deviations, ranges) and checked for normal distribution. As inferential statistics, we used *t*- tests, univariate and multiple regression analyses and a mediation analysis. We selected the following control variables: baseline depression-severity, age, gender, number of co-diagnoses in the final report, enhancement of inpatient antidepressant treatment and treatment duration. In all analyses, we considered confidence intervals (95%) and effect sizes. In *t*- tests, we computed Cohen’s *d* as effect size measure. For interpretation, we referred to Cohen [28], thus indicating effect sizes between 0.2 and 0.5 as low, effect sizes between 0.5 and 0.8 as medium and effect sizes above 0.8 as large [28]. In regression analyses, we computed Cohen’s *f*^2^ as effect size measure. *f*^2^- values between 0.02 and 0.15 indicate a low effect, values between 0.15 and 0.35 a medium effect and values above 0.35 a large effect [28]. For all analyses, we used IBM SPSS Statistics Version 25 [29]. For calculation of Cohen’s *d* and the related confidence intervals, we used the tool from Lenhard and Lenhard [30]. The effect sizes and the related confidence intervals in regression analyses were calculated by the *f-square Effect Size Confidence Interval Calculator* by Soper [31]. The mediation analysis based upon the approach of Hayes [32], which calculated direct and indirect effects by regression analyses. We used the SPSS-macro PROCESS of Hayes [32] to compute these direct and the indirect effects. Bootstrapping with *n* = 5000 iterations was testing the significance of the indirect effect [33]. For all other analyses, the statistical significance level was *p* < 0.05.

## Results

### Study realization and sample

Across 5 months, sixty adult inpatients (47.30 ± 12.82 years old; 58.3% females) were included into the intention-to-treat-analysis (Fig. 1*)*. All baseline self-reports returned between day 4 and day 6 postadmission. The period between baseline measure and study endpoint was 30.94 ± 17.68 days (Table 1). Five subjects dropped out (Fig. 1). Table 1 shows the baseline characteristics of the intention-to-treat-sample as well as those of the (per protocol) completer sample. There were no significant differences between these samples´ baseline characteristics (Table 1), ruling out relevant attrition bias of the study results by the drop-outs.

**Table 1:** Baseline Characteristics

	Intention-to-treat-sample		Completer-sample		
	*M*	(SD)	*M*	(SD)	*p*
Total (*n*)	60	–	55	–	–
Drop-out (*n*)	5	–	0	–	–
Age	47.30	(12.82)	48.13	(12.62)	0.73^a^
Gender:					0.70^b^
Male	25	–	21	–	
Female	35	–	34	–	
Study duration (days)	30.94	(17.68)	32.09	(17.46)	0.27^a^
BDI-FS-score (points)	8.29	(5.04)	8.61	(4.88)	0.73^a^
Diagnoses (*n*):					0.60^b^
Psychiatric	2.50	(1.30)	2.55	(1.32)	
Somatic	1.67	(1.74)	1.58	(1.76)	
Antidepressants (*n*)	1.35	(0.52)	1.36	(0.52)	0.89^a^

^a^*t*-test, ^b^Chi^2^-tests; *p* < 0.05

**Fig. 2:**
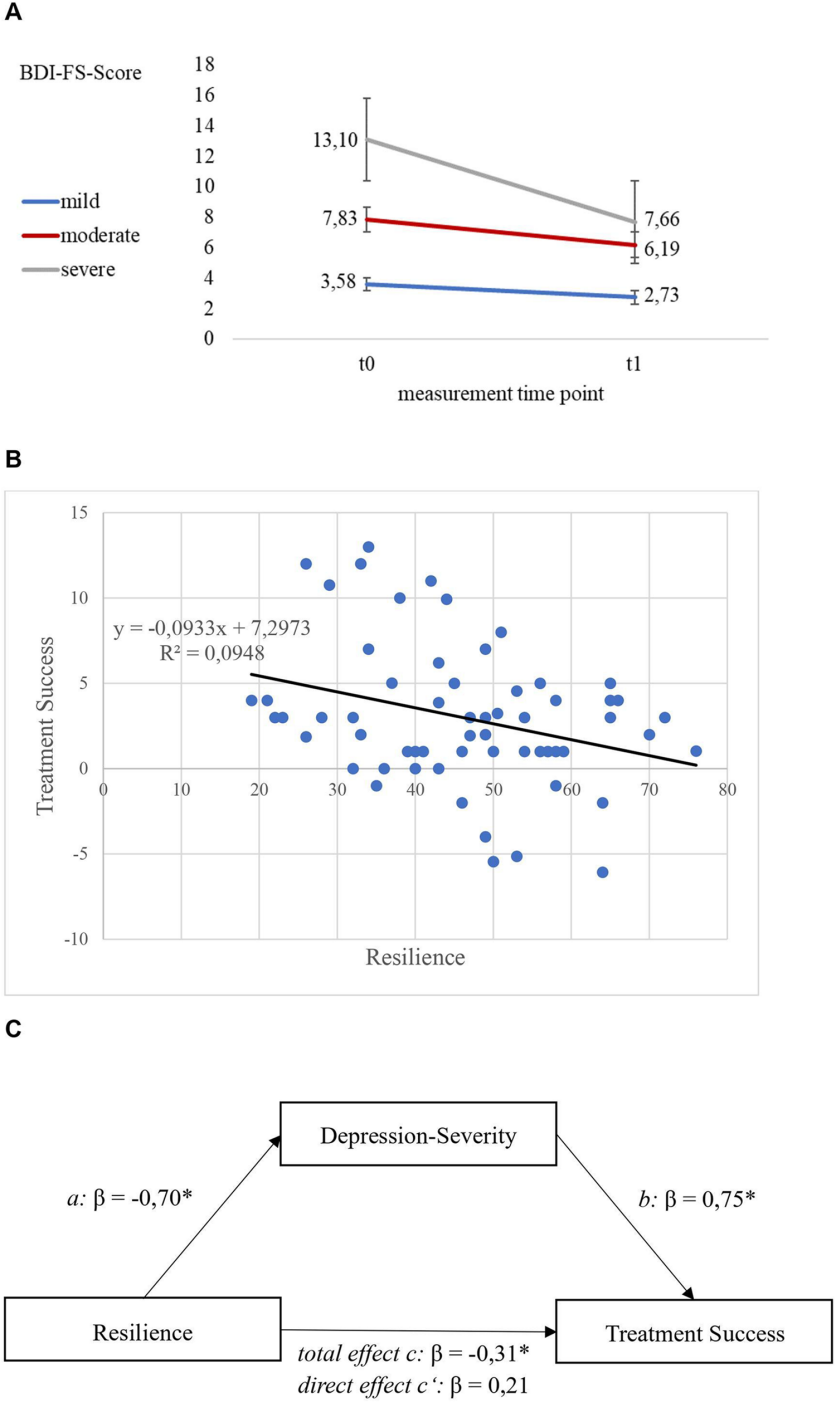
Treatment success (TS) depending on the subjective severity of MDD and resilience at baseline. **A** The stronger the self-perceived depression at baseline (t0), the stronger the TS at t1. Cut offs of the BDI-FS: no/mild (< 7 points, *n* = 27), moderate (7–9 points, *n* = 6), severe (> 9 points, *n* = 27) depression [20]. Only the TS of patients with severe MDD was significant (see Supplemental Table 2). **B** Resilience negatively predicted TS significantly (*p* = 0.017). The shared explained variation (*R*^2^) was 9.5%. (C) Mediation analysis: negative relationship between resilience and TS was significantly and fully mediated by the self-perceived depression-severity according to BDI-FS at baseline (*b* = − 0.16; 95%-CI [− 0.24; − 0.09]; *β* = − 0.52). *p < 0.05. Standardized regression coefficients: *β* > 0.1 = mild, *β* > 0.3 = moderate, *β* > 0.5 strong effect size [28]

All patients had used antidepressant (AD) pharmacotherapy prior to admission, which was directly enhanced by switching (*n* = 10) or augmentation (*n* = 46), left unchanged (*n* = 2) or reduced alongside the study (*n* = 2). According to the baseline BDI-FS-scores, twenty-seven, six, and twenty-seven patients fell into the category “no/mild”, “moderate” and “severe” depression, respectively (Fig. 2A). Please note, that this subjective baseline depression- severity was not measured directly upon admission, but between days four and six postadmission.

### Impact of resilience and concept-of-illness on treatment success

Both, the resilience and the concept-of-illness were normally distributed (Shapiro–Wilk- test: 0.99; *p* = 0.78 for resilience, and 0.98; *p* = 0.26 for concept-of-illness). The descriptive study results are given in *Supplemental Table 1*. We found that the more severe the patients had perceived their depression at baseline, the better was their treatment-success at study endpoint (Fig. 2A, *Supplemental Fig. 1 and Supplemental Table 2*).

Resilience (RS-11) turned out to be a negative predictor of the treatment-success (*b* = − 0.09; 95%-CI [− 0.17; − 0.02]; *p* = 0.017), with a small effect size (*f*^*2*^ = 0.11; [− 0.04; 0.30]). The regression line and equation are shown in Fig. 2* B*. The RS-11 was not associated with the concept-of-illness measure (*b* = 0.02; [− 0.02; 0.06]; *p* = 0.34) which itself did not influence the treatment-success (*b* = − 0.08; [− 0.59; 0.43]; *p* = 0.76). Among all seven concept-of-illness-dimensions, only ‘*trust in doctors*’ significantly depended on RS-11 (*b* = 0.02; [0.003; 0.034]; *p* = 0.02). This means, higher the baseline resilience, the higher the trust in doctors, with a small effect size (*f*^2^ = 0.10; 95%-CI: [− 0.04; 0.28]) and without significant impact on the treatment-success by this concept-of-illness-dimension. The other concept-of-illness-dimensions also failed to influence the treatment- success significantly.

Age, gender, inpatient AD- treatment, study duration, and number of co-diagnoses did not influence the treatment- success (Supplemental Table 3). When controlling for baseline depression-severity, resilience did no longer influence the treatment- success significantly (*b* = 0.07; [− 0.02; 0.15]; *p* = 0.15).

### Mediation analysis

The mediation analysis corroborated that the negative influence of the resilience on the treatment-success was completely mediated by the depression- severity at baseline (Fig. 2C). This means, high baseline resilience levels predicted a poor treatment- success at study endpoint, because patients with high resilience levels showed low depression levels at baseline, which per se were characterized by marginal or no relevant treatment- success (Fig. 2A, *Supplemental Table 2*).

## Discussion

Inpatients’ resilience was found to be negatively associated with the success of a 5 weeks lasting MDD- treatment. This relationship was fully mediated by the patients’ subjective depression severity at baseline. Patients who felt more severely depressed at baseline, experienced the best treatment success at study endpoint, which was in line with the previous investigations [34]. Remarkably, these persons showed the lowest resilience. This would support the reputation of resilience as a protective trait for MDD. However, we did not perform a second resilience measure at study endpoint which would have allowed to consider its conceptualization as state marker, too [35–38]. In this context, it should be mentioned that there is increasing evidence of a dynamic interplay between both trait (enduring) and process/developmental (state, temporary) conceptualizations of resilience [35–38].

The other personal resource, the concept-of-illness, was not associated with the treatment success or resilience. This contradicted our assumption that resilience and concept-of-illness might significantly intertwine or overlap in the treatment of MDD. A closer look revealed that among all seven concept-of-illness-dimensions, only the ‘trust in doctors’ significantly depended on RS-11: the higher the resilience, the higher the trust in doctors [39], but without significant impact on treatment- success by this concept-of-illness-dimension. The other concept-of-illness-dimensions also failed to influence the treatment- success.

The effect size of the influence of resilience on the treatment- success was low. However, studies on the significant relation of resilience and self-reported emotional health showed a wide range of effect sizes, including a considerable portion of low ones (e.g. [25, 26]) which, nonetheless, should not be denied for pointing to clinically relevant trends. In case of our study, we are surprised about a good portion of patients (*n* = 27) obviously improving within the first week after admission to minimal subjective depression levels. This beneficial development appeared to be principally related to high resilience levels of these patients (see below).

The present study has limitations due to the broad fundamental definitions of both, the resilience concept and the concept of illness [7, 10, 11, 14]. In addition, we could not exclude that beneficial or tolerance effects basing upon new medications became mainly apparent after the end of the study period. The same applied to the psychotherapeutic treatment. The pharmacological enhancement of the antidepressant therapy did not influence the treatment- success. The impact of non-pharmacological treatments on the treatment- success was not studied. Five patients dropped out of our study; all had terminated inpatient treatment prematurely. For what reasons was not documented. The baseline characteristics in Table 1, however, were not significantly different between the intention-to-treat- and completer population which reduces the role of drop-outs as relevant confounders. Regarding the considerable variance in study duration (Table 1), we could not claim that each participant followed a comparable treatment plan. However, the study duration itself did not influence the treatment- success. Furthermore, only self-ratings have been evaluated, which might have served also as an advantage, as highly subjective relationships were studied here. On the other hand, depressive participants might have underestimated their resilience and positive concept-of-illness due to negative cognitive bias immanent to the depression state. A guiding through the questionnaires by interview would have produced additional bias. Although the patients sought inpatient treatment due to considerable burden of suffering and had been resistant to prior outpatient MDD- treatment, of whom 45% (*n* = 27) rated themselves as being maximally mildly affected according to the BDI-FS at baseline. This raises potential concerns about whether the BDI-FS was actually an appropriate measurement describing the “true” burden of MDD in our patients. Saying that we have done our best to keep the study as objective as possible. In this context, this finding shed further light on the cognitive biases these patients are prone to and might reflect the power of non-pharmacological “healing” factors (e.g., care pt.’s receiving in the inpatient unit, pt. being the center of attention, pt.’s positive expectation/placebo effect of the new treatment) [6, 40, 41]. Nevertheless, as the baseline-measure was not performed directly at admission, but four to 6 days later, an “early sudden gain” [42] might have occurred, e.g., by changing from home to inpatient conditions. Our results would support the assumption that a stronger resilience might serve as a proxy of the development of an early MDD- improvement during the inpatient treatment.

### Potential implications/future directions

*Clinical practice:* Our (negative) results invite further inquiry into how to design effective interventions for patients with varying degrees of severity and levels of coping resources, including resilience. Nevertheless, the resilience measure might help to differentiate persons who were more prone to respond to psychiatric inpatient treatment from those people who would develop spontaneous early relief within a few days post-admission, which, however, warrants future study guided by this hypothesis. The results of this study, however, suggest that the latter persons (showing higher resilience levels at baseline) did not benefit from a longer inpatient MDD- treatment than 1 week.

*Clinical research:* For randomized, placebo-controlled inpatient studies, the intervention in question might start 1-week post-admission to reduce the impact of “early sudden gains” on the interventions’ placebo power [6, 40, 41]. In some well-controlled clinical studies, this is taken into account as “run-in period”, i.e., the period before the trial is commenced. On the other hand, we neglected a “pre-discharge stress”, i.e., the anticipation of leaving the patient role and having to manage their daily life and relationships again which often leads to a worsening of symptoms approximately 1 week prior to discharge. Hence, both baseline and follow-up measurements coincided with phases of the inpatient trajectory that distort measures of treatment success, which should be considered in future prospective longitudinal studies on outcome predictors. To address these biases, measurements should be also taken prior to admission (e.g., with the referring psychiatrist) and following discharge (with the attending psychiatrist).

Those with high resilience and less severe depression benefited less from the routine inpatient treatment program than those with low resilience and higher BDI-FS scores. Their relatively positive starting point automatically reduces the differential they can achieve in improving, compared to those with more severe symptoms and fewer personal resources such as resilience. To reduce those “ceiling effects” in measuring treatment effectiveness, it might be an option to include only patients within a certain severity range at baseline, to have a comparable study population.

We found that subjective illness models (as measured by concept-of-illness [14]) did not affect treatment effectiveness. Other than hypothesized, concept-of-illness did not depend on resilience, except for the dimension “trust in doctors”. This may reflect a certain lack of conceptual clarity. Although patients’ subjective illness models have been found in inform help- seeking, treatment success depends on shared subjective illness models between patients and doctors. This would mean that both parties to the treatment relationship come to reach a consensus over what the problem is, where it comes from and what should be done about it towards which desired outcome [43, 44]. Future studies could include shared measures of subjective illness representation (concept-of-illness) of patient- provider dyads. This would also reduce biases emerging due to the therapists’ treatment expectations. Patients entering psychiatric in-patient treatment with optimistic treatment expectations might be confronted with psychiatrists who may have developed, over the years of experience, a—what they see as—“realistic” view as to what extent patients are likely to improve or not. As there is a certain selection of who receives inpatient treatment towards the more severe cases, patients may experience these “realistic” expectations as a “negative prognosis” which undermines their own hope and optimism, and thus their resilience [45, 46].

Apart from these methodological lessons learnt from the limitations of the present study; future study might explore the impact of self-regulation mediators (e.g.., self-concept, self-belief, self-efficacy; being all very closely interrelated) on the treatment- success of persons with mental disorders [15, 47, 48] – if an inherent “Self” exists at all (e.g., [19, 49].

We propose adopting a personalized medicine approach also within psychiatric diagnostic and treatment processes (e.g., by assessing patient- resources such as resilience) and beyond genetic profiling and medication matching.

## Conclusion

Within the limitations of this study, we corroborate the reputation of resilience as a protective factor for MDD although resilience was found to be a (weak) negative predictor for inpatient treatment-success of MDD. In addition, we suggest a subjective “early sudden gain” against the MDD-burden within the first week post-admission in nearly the half of the inpatients who remarkably were also characterized by the highest levels of resilience. These “high-resilience”-patients might benefit best from a shorter period of inpatient MDD- treatment. The subjective illness representation of the participants had no impact on resilience or subjective treatment effectiveness.

## Supplementary Information

Below is the link to the electronic supplementary material.

Supplementary file1 (PDF 103 kb)
