# Supplementary material for: Do patients’ resilience and subjective illness representation predict the outcome of a routine inpatient treatment program of major depressive disorder?
Source: Eur Arch Psychiatry Clin Neurosci. 2021 Jun 30;271(7):1309–17. doi: 10.1007/s00406-021-01285-5 (PMC8429153; doi:10.1007/s00406-021-01285-5)
Supplement: Supplementary file 1 — Supplementary file1 (PDF 103 kb) [file 406_2021_1285_MOESM1_ESM.pdf]

Supplementary Material

Do Patients´ Resilience and Subjective Illness Representation Predict the Outcome of a Routine Inpatient Treatment Program of Major Depressive Disorder?

Laura Marschollek,[1] Udo Bonnet[1,2]

[1]*Department of Psychiatry, Psychotherapy and Psychosomatic Medicine, Evangelisches Krankenhaus Castrop-Rauxel, Castrop-Rauxel, Germany, Academic Teaching Hospital of the University of Duisburg-Essen, Essen, Germany*

[2]*Department of Psychiatry and Psychotherapy, Faculty of Medicine, LVR-Hospital Essen, University of Duisburg-Essen, Essen, Germany*

Corresponding author: *Udo Bonnet, Department of Psychiatry, Psychotherapy and Psychosomatic Medicine, Evangelisches Krankenhaus D 44577 Castrop-Rauxel, Grutholzallee 21, Castrop-Rauxel, Germany,* Email: udo.bonnet@uni-due.de

Supplemental Figures and Tables

Figures

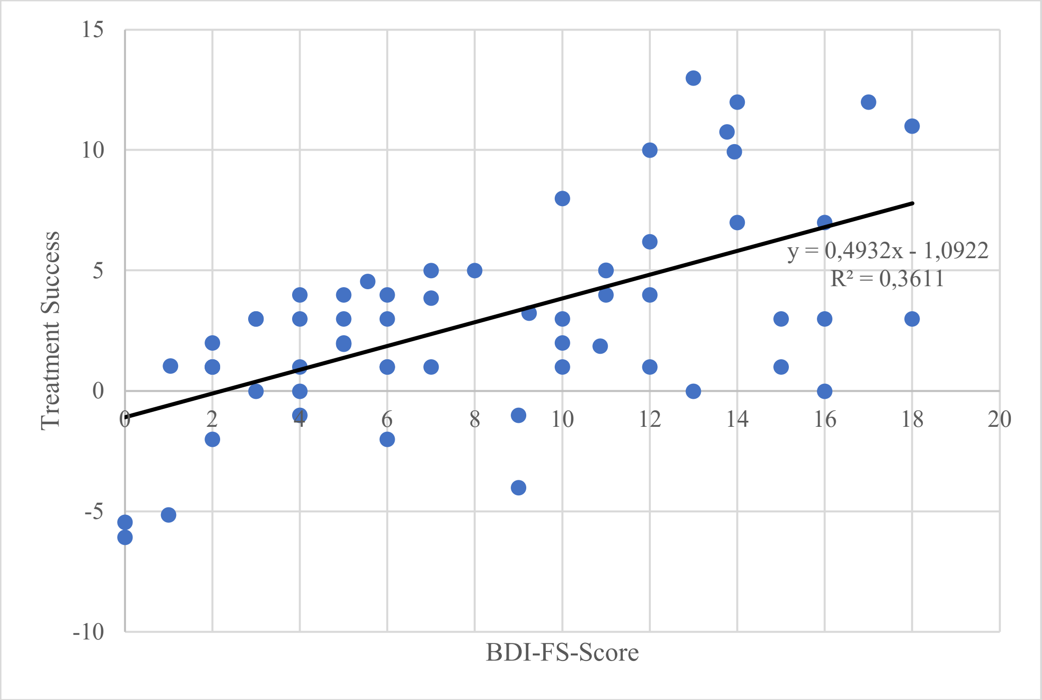

Supplemental Figure 1: Influence of Baseline Depression-Severity on Treatment-Success.

Tables

Supplemental Table 1: Descriptive Statistics

	M	*(SD)*	95%-CI	
			Lower	upper
Resilience	46.08	(13.65)	42.55	49.60
Concept-of-Illness	16.18	(2.00)	15.66	16.69
BDI-FS Baseline	8.29	(5.04)	6.99	9.59
BDI-FS Study Endpoint	5.29	(4.18)	4.21	6.37
Treatment-Success*	3.00	(4.14)	1.93	4.07

*Δ BDI-FS Baseline – BDI-FS Study Endpoint

Supplemental Table 2: Differential Treatment-Success (Δ BDI-FS Baseline – BDI-FS Study Endpoint) depending on the depression severity at baseline (t-tests)

Depression-Severity	Δ t0 – t1 [95%-CI]	Df	*T*	p	Cohens *d* [95%-CI]
No/mild *	0.85 [-0.29; 1.99]	26	1.53	0.14	0.41 [-0.11; 0.92]
Moderate	1.64 [-2.19; 5.48]	5	1.10	0.32	0.51 [-0.01; 1.03]
Severe	5,44 [3.83; 7.06]	26	6.95	< 0.01	1.55 [0.97; 2.13]
Sum	3.00 [1.93; 4.07]	59	5.61	< 0.01	0.65 [0.13; 1.17]

Cut offs of the BDI-FS: no/mild (< 7 points, n = 27), moderate (7-9 points, n = 6), severe (> 9 points, n = 27) depression [20]. *There were 12 patients showing at baseline a BDI-FS score below 4 points indicating no subjective depression. This subgroup developed a slightly negative treatment-success (mean -0.55, SD 3.29 points) at study endpoint. Patients with mild depression (n = 15) developed a positive treatment-success (mean 1.97, SD 1.96 points) at study endpoint.

Supplemental Table 3: Influence of control variables on the treatment-success

Control variable	*b*<br>[95%-CI]	p	*f²*<br>[95%-CI]
Age	0.004<br>[-0.065; 0.072];	0.914	0.001<br>[-0,013; 0,016]
Gender	0.327<br>[-1.571; 2.225]	0.732	0.001<br>[-0.014; 0.016]
Enhancement of ADT*	-1.341<br>[-4.681; 2.000]	0.425	0.075<br>[-0.045; 0.217]
Study Duration	-0.018<br>[-0.048; 0.011]	0.221	0.016<br>[-0.041; 0.080]
Number of Co-Diagnoses	0.109<br>[-0.627; 0.844]	0.768	0.001<br>[-0.013; 0.016]

*switch, augmentation; ADT = antidepressant treatment
